# B Cell Activation Triggered by the Formation of the Small Receptor Cluster: A Computational Study

**DOI:** 10.1371/journal.pcbi.1002197

**Published:** 2011-10-06

**Authors:** Beata Hat, Bogdan Kazmierczak, Tomasz Lipniacki

**Affiliations:** 1 Institute of Fundamental Technological Research, Polish Academy of Sciences, Warsaw, Poland; 2 Rice University, Department of Statistics, Houston, Texas, United States of America; La Jolla Institute for Allergy and Immunology, United States of America

## Abstract

We proposed a spatially extended model of early events of B cell receptors (BCR) activation, which is based on mutual kinase-receptor interactions that are characteristic for the immune receptors and the Src family kinases. These interactions lead to the positive feedback which, together with two nonlinearities resulting from the double phosphorylation of receptors and Michaelis-Menten dephosphorylation kinetics, are responsible for the system bistability. We demonstrated that B cell can be activated by a formation of a tiny cluster of receptors or displacement of the nucleus. The receptors and Src kinases are activated, first locally, in the locus of the receptor cluster or the region where the cytoplasm is the thinnest. Then the traveling wave of activation propagates until activity spreads over the whole cell membrane. In the models in which we assume that the kinases are free to diffuse in the cytoplasm, we found that the fraction of aggregated receptors, capable to initiate B cell activation decreases with the decreasing thickness of cytoplasm and decreasing kinase diffusion. When kinases are restricted to the cell membrane - which is the case for most of the Src family kinases - even a cluster consisting of a tiny fraction of total receptors becomes activatory. Interestingly, the system remains insensitive to the modest changes of total receptor level. The model provides a plausible mechanism of B cells activation due to the formation of small receptors clusters collocalized by binding of polyvalent antigens or arising during the immune synapse formation.

## Author Summary

B cells are activated in response to binding of appropriate ligands, which induces the aggregation of B cell receptors. The formation of even small clusters containing less than 1% of all the receptors is sufficient for activation. This observation led us to a model in which the receptor cluster serves only as a switch that turns on the activation process involving also the remaining receptors. The idea of the model exploits the fact the Src kinase - BCR system is bistable, and thus its local activation may start the propagation of a traveling wave, which spreads activation over the entire membrane. We found that the minimal size of the activatory cluster decreases with the thickness of the cytoplasm and kinase diffusion coefficient. It is particularly small when kinases are restricted to the membrane. These findings are consistent with the properties of B cells, which prior to activation have extremely thin cytoplasmic layer and in which Src family kinases (interacting with the receptors) are tethered to the membrane.

## Introduction

B lymphocytes activation is initiated by B cell receptor (BCR) aggregation following antigen engagement. Activated B cells can differentiate to form extrafollicular plasma-blasts responsible for the rapid antibody production and early protective immune responses. Alternatively, they can differentiate into plasma cells, which can secrete high-affinity antibody, or memory B cells, which provide long-lasting protection [1].

BCR is composed of the highly varied membrane-bound immunoglobulin (mIg) molecule and a heterodimer of the Igα and Igβ chains containing the tyrosine-based motifs (ITAM) which can be phosphorylated by members of the Src family kinases (SFKs) [2]. In turn, phosphorylation of ITAMs enables the stable binding of SFKs via SH2 domains, preferentially to Igα chains, which are then activated by transphosphorylation, see [3] for review. When B cell receptors are aggregated, the weakly-bound Src kinases initiate phosphorylation of the ITAMs of neighboring receptors. The phosphorylated tyrosines of Igα chain ITAM then bind more stably to the SH2 domains of the Src kinases, thus allowing the kinases to mediate phosphorylation of both Igα and Igβ chains more efficiently [4]. The higher affinity SH2 domain binding enables Src kinase transphosphorylation in the activation loops thereby increasing their catalytic activity [5]. Thus, although BCR does not directly activate Src kinase, binding of Src kinases to phosphorylated ITAM motifs enables Src kinase phosphorylation. The phosphorylated tyrosines of the Igβ ITAM recruit a cytosolic protein, tyrosine kinase Syk, which mediates phosphorylation of proteins acting further downstream in the signalling pathway [6].

We recently demonstrated by a computational analysis of a reaction-diffusion model that the strength of positive feedback controlling cell ability to be activated, is regulated by the kinase diffusion coefficient [7] and the spatial distribution of the membrane receptors [8]. Rapid kinase diffusion, although enhances transmission of activity towards cell nucleus, causes that the activated kinase quickly leaves the vicinity of the cell membrane, and cannot activate receptors. As a result, for a broad range of parameters the cell can be activated only if the kinase diffusion coefficient is sufficiently small [7]. Moreover, aggregation of receptors increases the chance that the receptor activated kinase will target the other (neighboring) receptors, before it will be dephosphorylated by phosphatases. We showed that aggregation of membrane receptors alone can trigger cell activation [8]. In this paper we explore a modified reaction-diffusion model of a mutual kinase-receptors interaction in the context of B cells. Below, we review the established facts of B cell receptor signaling, which led us to the considered model.

Upon the contact with antigen-presenting cell, the B cell membrane is reorganized, leading to the formation of an immunological synapse, which induces B cell spreading over the antigen-containing surface, and then its contraction [1], [9]. During the contraction phase the antigens are gathered into the central cluster, with an area of less than 10% of the total cell surface [10]. Formation of BCR clusters triggers rapid phosphorylation of BCRs and the associated SFK [11].

B cell activation follows the recognition of membrane-bound specific antigens by BCR [12]. Antigen binding drives the formation of BCR clusters that initiate the formation of signaling complexes consisting of BCRs and SFKs [13], [14]. These clusters can be formed by receptor cross-linking due to binding of polyvalent ligands recruited from the solution [14]. Alternatively, the receptor clusters can be formed by a B cell contact with an antigen-presenting cell (APC) loaded with antigens. It was showed by Batista et al. [15] and then by Tolar et al. [16] that monovalent ligands are also capable to initiate BCR signaling if presented on APC. Formation of the immunological synapse is a way to select the strongly binding antigens from the surface of the antigen presenting cell and to collect them into a smaller area. Large antigens, including viruses and immune complexes, are captured from the lymph by macrophages and are presented by these macrophages to B cells, see [17]. Dintzis and Vogelstein [18], [19] proposed a theory in which about 10–20 BCR rich aggregates (immunons) which are formed upon binding of the highly multivalent antigens, like viruses, are capable of triggering B cell responses. This idea was then theoretically investigated by Sultzer and Perelson [20], [21] who were able to explain the experimental dose-response curves, obtained with highly multivalent antigens mixed with ligands of lower valence. Summing up, in both modes of activation – by monovalent and polyvalent large antigens–the limiting step in B cell activation is the formation of BCR cluster. As discussed, these aggregates can be formed by direct cross-linking of receptors, or by diffusion trapping after kinetic segregation on the B cell membrane. Aggregates form independently of signaling through BCRs, because they were observed to form on membranes of Lyn deficient B cells following antigen stimulation [11]. Formation of microclusters leads to rearrangement of the corticalactin network and formation of corrals around BCR microclusters. These fibroactin corrals are responsible for restricting BCR diffusion and maintaining integrity of BCR microclusters [11].

Src family includes at least eight highly homologous proteins [22]; the three SFKs most abundantly expressed in B cells are Lyn, Blk and Fyn [23]. As shown by Sato et al. [24], Src kinases trafficking is specified by the palmitoylation state. Non-palmitoylated SFK like Blk rapidly move between the plasma membrane and late endosomes or lysosomes, mono-palmitoylated Src kinases, such as Lyn are transported to the cell membrane via the Golgi apparatus, whereas dually palmitoylated Fyn, is directly targeted to the membrane [24]. Sato et al. examine the localization of SFKs by transfecting monkey kidney epithelial cells (COS-1) with Lyn and Fyn. They found that in the early phase of expression (8 hours), Lyn is predominantly localized in the perinuclear region and gradually moved to the plasma membrane in the later phase (24 hours), while the majority of Fyn was found at the plasma membrane from the early phase. In some cell lines the transient nuclear localization of SFKs was observed: Fyn in zebrafish embryo development [25], Lyn in HeLa cells with Lyn inhibitor Csk overexpression [26]. Recently, Takahashi et al. reported nuclear localization of SFK in COS-1 cells [27]. Nevertheless, SFKs lack nuclear localization signal (based on http://pprowler.itee.uq.edu.au/NucImport, [28]) and majority of studies indicated that in immune cells SFKs predominatly localize either on the cytoplasmic face of plasma membrane or transiently in the perinuclear region [29],[24].

The cytoplasmic kinase Syk binds preferentially to doubly phosphorylated Ig*β* chains, where it is transphosphorylated. Rolli et al. [30] found that Syk also mediates positive feedback phosphorylating ITAM motifs. In particular, they demonstrated that Lyn phosphorylates ITAMs only at the first tyrosine residue, whereas Syk phosphorylates both tyrosines of the ITAM. Furthermore, Syk is a positive allosteric enzyme which is strongly activated by binding to the phosphorylated ITAM tyrosine residues. Thus, the recruitment of Syk to the phosphorylated ITAM allows Syk to catalyze the phosphorylation of more ITAMs allowing the recruitment of additional Syk molecules to the clustered BCR complexes [31]. This leads to further amplification of the signaling [32].

Since in the proposed model all receptor interacting kinases are represented by a single kinase species, with respect to the above properties of Src kinases and Syk, we will consider two cases:

1. in which the receptor interacting kinase diffuses in the cytoplasm
2. in which the kinase is tethered to the cell membrane.

B cell activation leads to irreversible cell fate decisions and differentiation into cells capable of secretion of protective antibodies or memory cells that provide long-lived protection against secondary infection (see [11] for review). One can thus expect that this regulation is associated with bistability [33]. In particular, Bhattacharya et al. proposed that B cell differentiation relies on bistable switch [34]. Bistable systems are capable to convert the graded signals into well defined *all-or-nothing* responses [35], [36]. They are typically associated with positive feedback loops and nonlinearities [37]. The considered system of Src kinases interacting with BCRs has several positive regulatory couplings. In particular

1. Src kinases phosphorylate ITAM motifs of Ig*α* and Ig*β* chains, in turn phosphorylated ITAM stably bind Src kinases
2. Src kinases bound to ITAM phosphorylate one another
3. Syk binding requires ITAM phosphorylation, which in turn is mediated at least partially by Syk. Phosphorylation of ITAM tyrosines by Syk provides a positive feedback loop [32].

As discussed above, the formation of a receptor cluster is the limiting step in initiation of the signaling. In this study we propose a reaction-diffusion model which can explain how signal initiated by a small BCR cluster can propagate and trigger activation of the remaining receptors. In the considered model we omit the step of ligand binding and the process of receptor aggregation. Instead we assume, according to the above discussion, that as a result of the ligand binding, some fraction of receptors is aggregated and immobilized in a portion of the cell membrane.

The model exploits the assumption that the BCR-SFK regulatory system is bistable. Fuss et al. proposed that the SFK activity is controlled through a bistable switch resulting from SFK kinase autophosphorylation [38], [39]. Next, Kaimachnikov and Kholodenko [40] showed that SFK can display oscillatory, bistable and excitable behaviors. They demonstrate that the saturability of phosphatase activity suffices for bistability. In the model proposed in this study, bistability arises due to Michaelis-Menten kinetics of SFK dephosphorylation, and in addition due to distributive phosphorylation of ITAM tyrosines. As discussed in [27], the distributive phosphorylation requires sufficiently large diffusion. For small diffusion and short reactivation time (the time needed for the kinase to release ADP and bind the next ATP molecule), the rapid rebindings of the enzyme can turn the distributive phosphorylation into the processive one. Each of these two nonlinearities suffices for bistability, but their combination causes that the bistability range is broader. Bistable, spatially extended systems may be triggered by a relatively small but localized signal which induces local transition from inactive to active state. Thereafter, the surge of kinase and receptors activity propagates as a traveling wave. Relevant to our model, Wang et al. observed mechanically initiated Src activity wave propagating over plasma membrane [41].

We will show that the minimal size of the activatory cluster decreases with decreasing the thickness of the cytoplasm-which is very scanty for B cells. The matured B cells (that range in size from 7–20 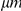) frequently retain the original 4∶1 nuclear-cytoplasmic volume ratio of premature cells which gives the ratio of the nuclear to cell radii of about 0.93 [42]. We will show also that cell can be activated in response to nuclear displacement without receptor clustering. In this case, the activation starts in the place where the cytoplasm is the thinnest, and then propagates over the rest of the cytoplasm. Nuclear movements during synapse formation are well documented experimentally in T lymphocytes [43].

## Models

In what follows, the dimensional variables and dimensional coefficients will be denoted with superscript ^*^, whereas in non-dimensional variables and coefficients superscript ^*^ will be omitted. First we will introduce the model in dimensional variables, next we will nondimensionalize it. The values of the coefficients, in their dimensional and non-dimensional forms are listed in Table 1.

**Table 1:** Parameters in the models.

Parameter	Description	Literature values	Values in the model	Nondimen. values in simulations
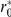	B cell radius	3.5−6*µm* [59],[60]	6*µm*	1
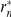	nuclear radius	0.93× 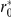 [42] a)	(0.8,0.9,0.95,0.98)× 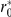	0.8,0.9,0.95,0.98
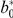	coefficient of receptor dephosphorylationb)	0.1−100*s* ^−1^ [51]	1*s* ^−1^	1
*b* ^*^	coefficient of kinase dephosphorylation	0.26−670*s* ^−1^ [40] c)	6−25*s* ^−1^	6−25
*d* ^*^	kinase diffusion	0.1−1 *µm* ^2^/*s* in cytosol [51], [61], [62]	36,4,0.36 *µm* ^2^/*s*	--------
		0.01−0.1 *µm* ^2^/*s* on membrane [60]	36,4,0.36 0.04,0.0013 *µm* ^2^/*s*	--------
α	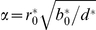	2−200 in cytosol	---------------	1 (Figs.3,4) 3 (Figs.3,4,S2) 10 (Figs.2-5,S2-S5)
		6−600 on membrane (based on literature values of 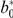 and *d*, for 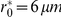 )	---------------	1 (Fig. 6), 3 (Figs.6,7), 10 (Fig.6), 30 (Fig.6 ), 167 (Fig.6)
*a* ^*^	coefficient of kinase phosphorylation	---------------d)	---------------	1 (cytosolic model) 3/( 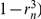 (membrane model)
*ρ* ^*^	coefficient of receptor phosphorylation	---------------d)	---------------	1
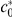	spontaneous receptor activation coefficient	---------------	---------------	0.01e)
*P* ^*^	surface density of receptors	200/*µm* ^2^ [60] 500/*µm* ^2^ [63] f)	---------------	1
*Q* ^*^	density of kinase	--------------------g)	---------------	1
*H*	nondimesional Michaelis coeff.	0.1–1 [40]	0.1	0.1

a) according to the measured ratio of the B cell nucleus volume *V_N_* to the B cell cytosol volume *V_C_*: 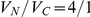 [42].

b) pseudo first order reaction; this coefficient determines the relative time scale.

c) estimated in [40] (online supplementary material) based on [64],[65], [66].

d) The SFK and BCR phosphorylation takes place at the membrane. The experimental data are scarce and provide only the coefficients of phosphorylation referred to the molar concentrations of kinase, which is not adequate for description of the processes on the membrane.

e) the value of *c*_0_ reflects the assumption that the activity of the unphosphorylated kinase is 100 times smaller than the unphosphorylated kinase; in [40] the ratio of unphosphorylated to phosphorylated kinase activity is assumed to be equal to 0.05.

f) the receptor surface density estimated basing on the number of receptors 2.5×10^5^ given in [63] and the surface of cell membrane *S* of radius 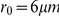 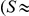
500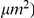 is approximately 500/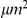.

g) Faeder et al. estimated the number of available Lyn kinase molecules as 2.8×10^4^ [67] The remaining Lyn is in its inactive, closed conformation in which kinase domain is inaccessible due to the intramolecular binding. Assuming that Fyn and Blk are present in a similar quantities as Lyn, the total amount of available SFK would be of order 10^5^. We are not giving here the molar density of SFK. As discussed SFK occupy only a small fraction of cell volume, thus the molar concentration referred to whole cell volume is a misleading quantity.

**Figure 1:**
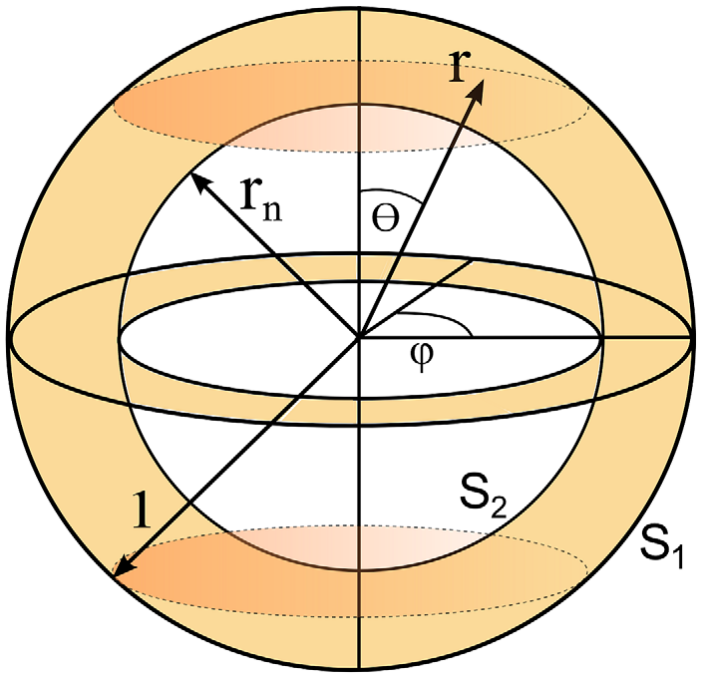
Geometric model of the cell. The spherical coordinates of a point will be denoted by *r*, 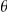 and 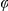. The shaded region corresponds to the cell cytoplasm and the unshaded to the nucleus.

The cell is modelled geometrically as a ball 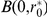 of radius 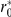. A concentric ball 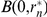 of a smaller radius 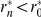 models the cell nucleus (Figure 1). The total concentration of the membrane receptors is denoted by 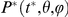, the concentration of active membrane receptors is denoted by 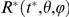, whereas the concentration of the active kinases, diffusing over the cell cytosol, is denoted by 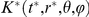. We assume that diffusion coefficients of active and inactive kinases are the same and equal to d^*^, and thus the total kinase concentration may be assumed constant in cytosol equal to Q^*^. It is assumed that there is no flux of the kinases through the nuclear membrane. The kinase is activated by active receptors on the cell membrane. The flux of the activated kinase is modeled by the nonlinear Robin type boundary condition and is proportional to the concentration of active receptors and the inactive kinase 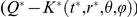 at the boundary. Receptors are activated by active kinases or “spontaneously” at some small rate. The spontaneous activation can be interpreted as the activation by inactive kinases or by some other kinase species. We assume that receptors are dephosphorylated at a constant rate equal to 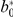 by uniformly distributed membrane phosphatases. As already said, we will neglect receptors diffusion and we will focus on the case in which fraction of them is aggregated in the cluster occupying a portion of the membrane. We also assume that phosphatases that dephosphorylate kinases are uniformly distributed. In such a case the pseudo first order kinase dephosphorylation reaction can be described by dephosphorylation rate parameter b^*^, Michaelis constant H^*^, and Hill coefficient equal to 1, and these coefficients may be assumed constant in time and space [44],[45]. Alternatively, one can use a more detailed Smoluchowski approach, that is frequently used when spatially non-uniform forces are present [46].

With respect to the above assumptions the active kinase concentration K^*^ within cytosolic domain 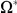 satisfies the reaction-diffusion equation: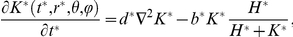 with the boundary conditions on the cell membrane 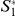 and nuclear membrane 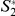


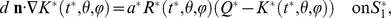


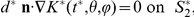
 where denotes the scalar product, and **n** denotes the unit vector normal to 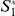 or 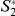 directed outward 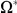, b^*^ is the coefficient of the phosphatase activity and H^*^ is the corresponding Michaelis constant. The concentration of active receptors R^*^ satisfies an ordinary differential equation on 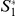 of the form: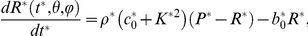 where 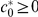 defines spontaneous activation of receptors. The system is supplemented by the initial conditions
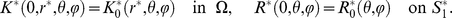



The nonlinearities in the model arise from the assumptions that the amount of the phosphatase is limited and the receptors require double phosphorylation for full activation. We will consider the axi-symmetric receptor distributions 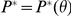 and associated axi-symmetric solutions.

Now we transform the above to a non-dimensional form. Let 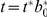 and 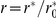. Then the cell corresponds to the ball 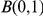 of radius 1, whereas the nucleus corresponds to the ball of radius 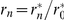, thus the cytosol region corresponds to 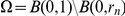. Let 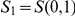 and 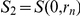. Then the considered system can be rewritten as
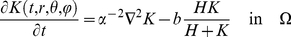
(1)

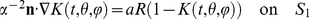
(2)

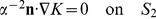
(3)

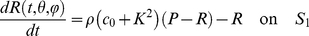
(4) with the initial conditions
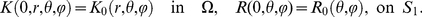
(5) where 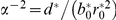 is the non-dimensional diffusion coefficient, 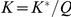, 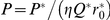, 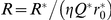, 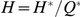, 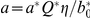, 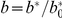, 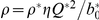 and 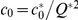, where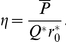 and 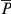 is the average surface density of receptors. Below, we will put 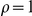 in the cytosolic model.

### Membrane model

In this variant of the model we assume that the kinase is tethered to the cell membrane. In such a case the 3D model may be replaced by a 2D model on the cell membrane. Moreover, if the axial symmetry is assumed the problem becomes essentially 1D, in which 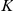 and 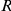 are the functions of *t* and 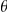 only, where 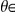 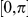. In the non-dimensional form the system of equations for membrane model reads:
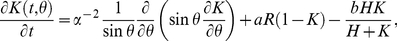
(6)

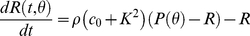
(7)

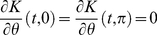
(8) with initial conditions
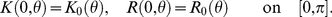
(9)


As in the cytosolic model, we will put 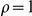. In the case of thin cytoplasm, i.e. when 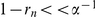 for 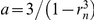 the solutions of the system (6)–(9) approximate the solutions of the original model (1)–(5) . In the original model the last condition implies that the kinase activity is almost constant on the depth of the cytoplasm, thus only the dependence of *K* on *θ* is important.

## Results

### Limit of infinite diffusion

In the limit of infinite diffusion (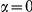) for 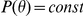 the system is perfectly mixed, thus the concentrations, *K* and *R*, are constant in space. In this case systems (1)–(4) and (6)–(7) converge to the system of ordinary differential equations
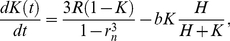
(10)

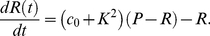
(11)


One can obtain the above system by integrating Eq. (1) over 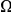 and using the Gauss theorem. The system (10)–(11) can be mono- or bistable, i.e. it has one or two stable stationary solutions depending on parameters *c*_0_, *H* and *B*, where
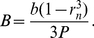
(12)


In the case of bistability, the two stable solutions correspond to the states with low and high levels of the active kinase and receptors. These states will be referred to as *inactive* and *active*. In Figure S1, we show the bistability regions in (*B*, *H*) plane for several values of the spontaneous activation constant 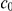. The bistability regions do not depend explicitly on 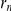, because this parameter is already incorporated in *B* defined in (12). The bistability range is the largest for 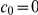 and shrinks with increasing 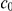, see Text S1.

In further analysis, in order to reduce the number of free parameters, we fix the spontaneous activation constant 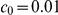 and the Michaelis constant *H* = 0.1. As may be deduced from Figure S1, such a choice of 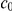 and *H* implies a robust bistability. As shown in Figure S1, the bistability region is even broader for 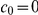, but since this is non-generic case and can lead to results valid only for the zero basal activity of kinases and receptors, we focus on the case of small but positive 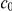.

**Figure 2:**
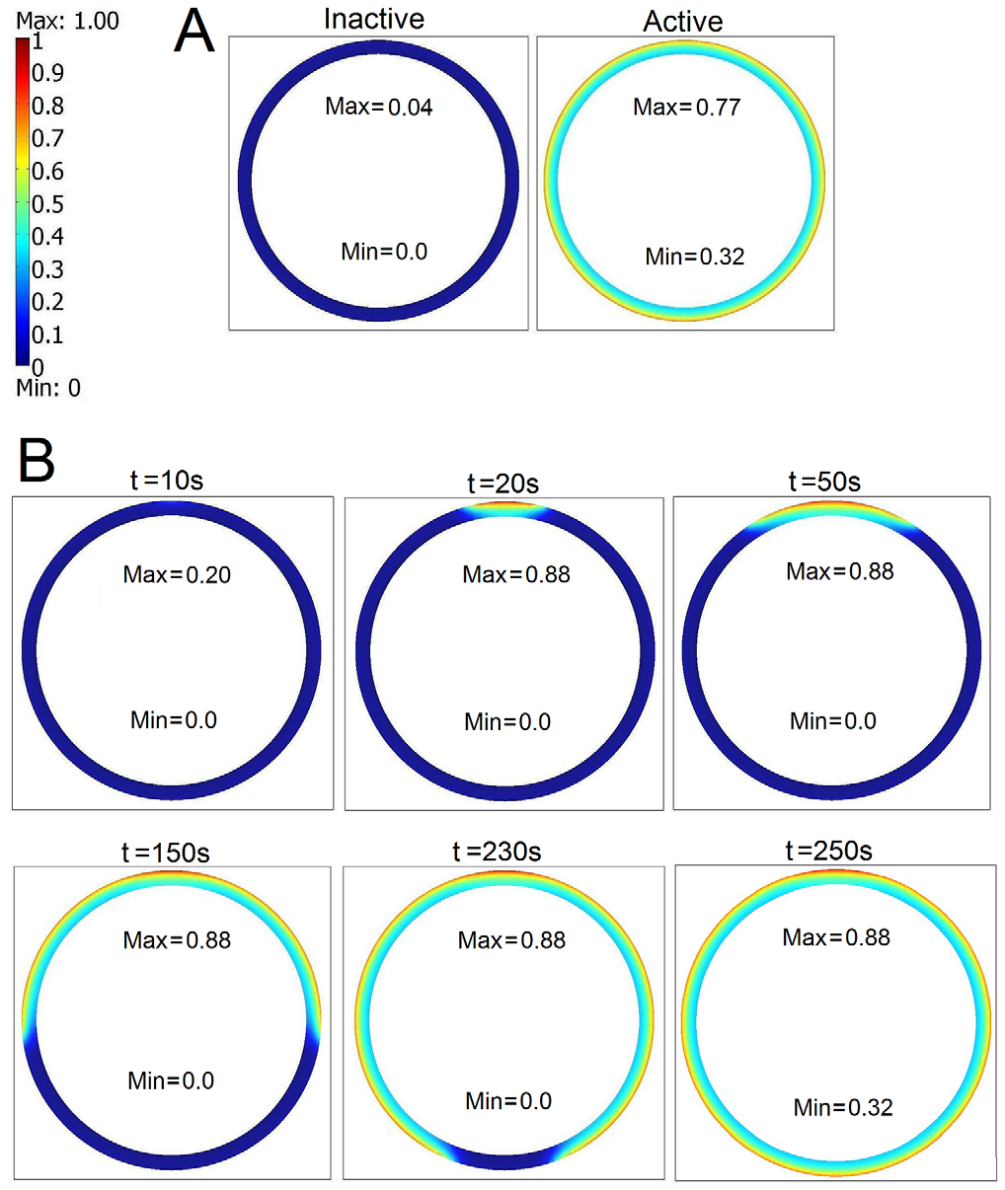
Visualization of the travelling wave solution. Panel A: two stable stationary solutions for 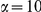, 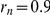, 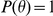 and 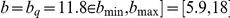. Panel B: propagation of the traveling wave connecting the two stable solutions shown at Panel A, initiated by a formation of a cluster of receptors at the upper cell pole. The aggregated fraction of receptors is *F* = 0.01 and the cluster size is 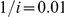. For *t* = 250 *s* the whole cell is activated. The minimum and maximum value of the active kinase concentration are given in each panel. In Panel A the active kinase distribution 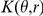 is spherically symmetric. In Panel B the active kinase distribution 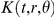 is not reaching the spherical symmetry even for *t* = 250 *s* the small cluster of receptors at the upper pole causes that locally the active kinase concentration is elevated with respect to spherically symmetric case (0.77 versus 0.88).

The numerical simulations leading to the results presented in Figures 2–7 and Figures S3–S6 were performed using the COMSOL multiphysics simulation software environment.

**Figure 3 pcbi-1002197-g003:**
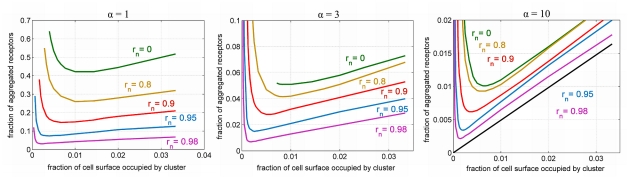
The dependence of the fraction of the aggregated receptors *F* on the fraction of cell surface 1/*i* occupied by the cluster for *r_n_* = 0, 0.8, 0.9, 0.95 and 0.98, for 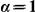, 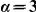, 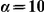.

**Figure 4 pcbi-1002197-g004:**
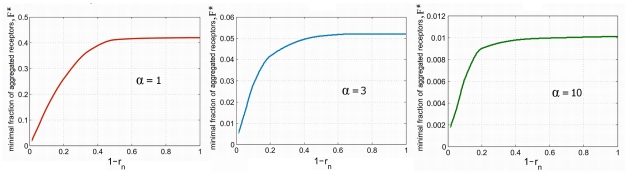
The dependence of the minimal fraction of the aggregated receptors sufficient for the cell activation 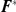, on thickness of the cytoplasmic layer (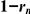) for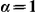, 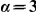, 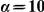.

**Figure 5 pcbi-1002197-g005:**
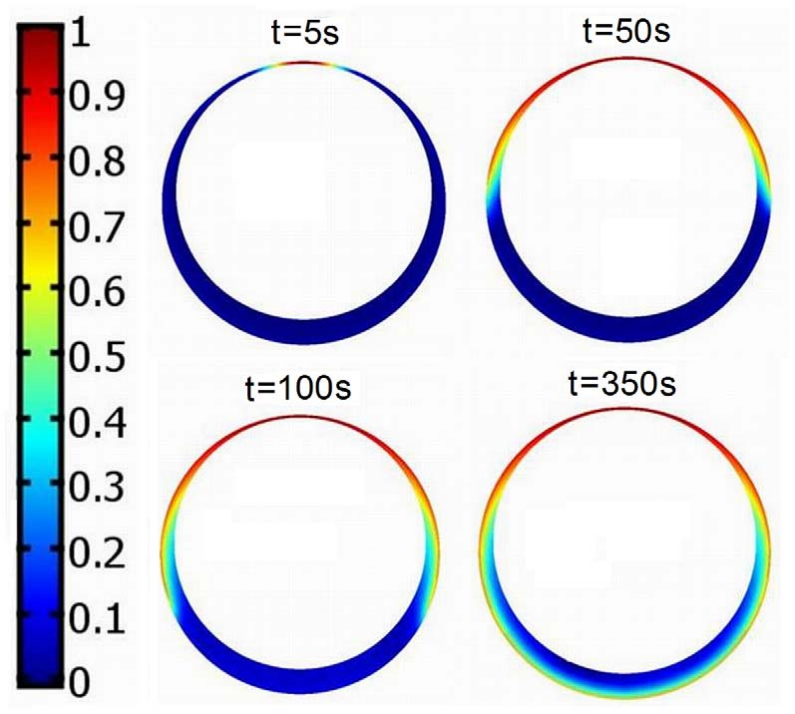
Activation of the cell by the displacement of the nucleus. The shift of the nucleus by 0.08 causes that locally thickness of the cytoplasm drops to 0.02. This leads to the local cell activation, thereafter the wave of the kinase activity spreads over the rest of the cytoplasm. The concentration of the active kinase is the highest at the pole in which the cytoplasm is the thinnest. The remaining parameters for this simulation are 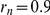, 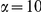, 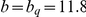.

**Figure 6 pcbi-1002197-g006:**
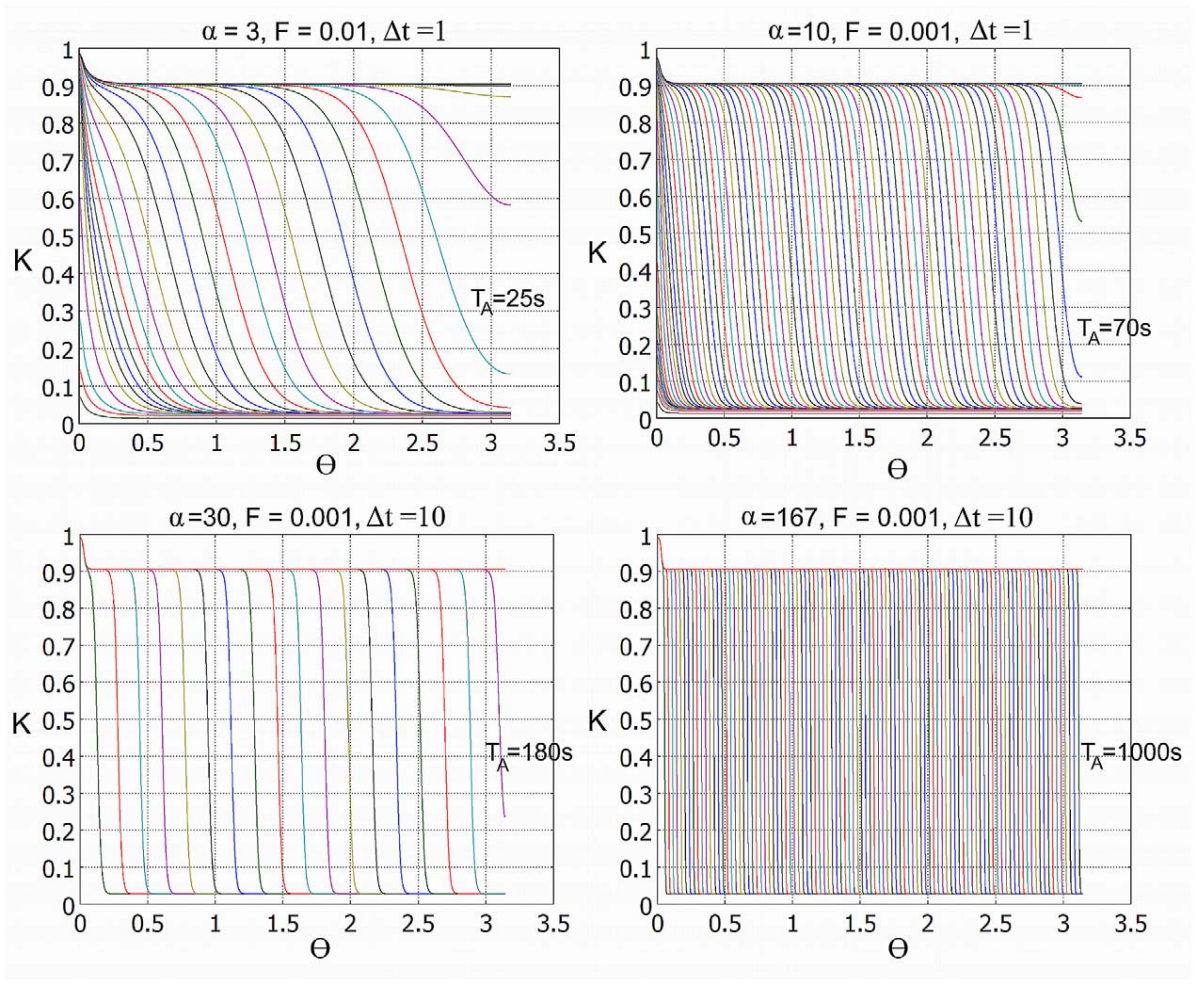
Time snapshots of the active kinase concentration 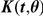. The initial condition is 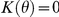, 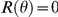. The traveling wave of activation is induced by a local aggregation of the receptors; 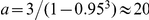, 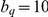, 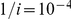. Panel A: 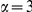, 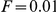, time interval is 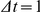, activation time 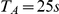; Panel B: 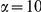, 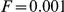, 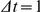, 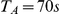; Panel C: 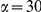, 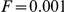, 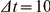, 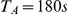; Panel D: 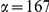, 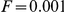, 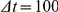, 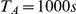.

**Figure 7:**
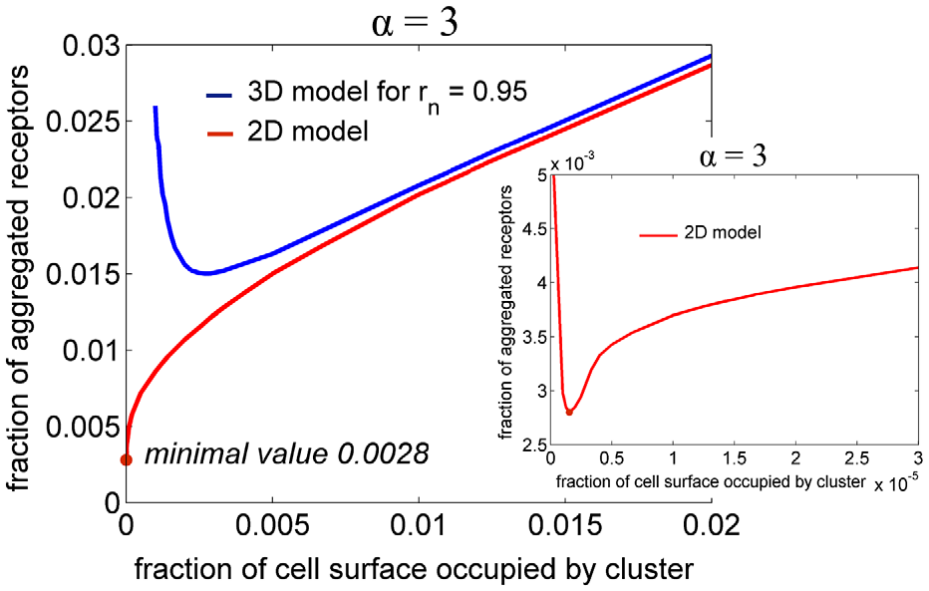
The dependence of the critical fraction of the aggregated receptors on the fraction of cell surface occupied by the cluster for 2D (red) and 3D (blue) models for 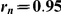 and 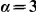. The minimal fraction of aggregated receptors 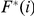 for 2D model equals 0.0032 at *i* = 5×10^5^. Right Panel: the zoomed region around point 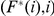 for 2D model.

### Cell activation in the cytoplasmic model

As shown in Figure S2 in a certain range of parameters the system is bistable also for finite diffusion. In the further considerations we focus on the particular bistable case. That is to say, for a given *α* and *r_n_* we choose such 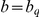, for which the system is bistable (for 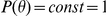) and loses its bistability (becomes monostable active), precisely when the receptor level increases to 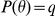 with all other parameters fixed. We set *q* = 1.5. Such a choice of *q* is arbitrary, but it does not qualitatively influence the system behavior. It assures that the system (for the reference receptor level 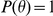) cannot be activated by a modest increase of the total receptor level. For example, for α = 0 and 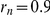 the system is bistable for 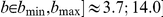 with 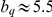, for α = 3 and 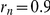 the system is bistable for 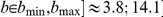, whereas 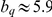. For 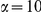 and 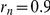 the system is bistable for 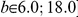 and 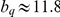. As shown in Figure 2, for α = 10, 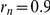, 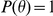 and 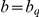 the system has two stable stationary solutions, inactive and active. The solutions starting from zero initial condition 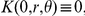 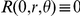 converge to the inactive state, whereas starting from initial condition 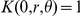, 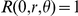 converge to the active state.

As discussed above, our choice of *b_q_* guarantees that the cell, when inactive, cannot be activated by a modest increase of the total receptor level, assuming that the receptors are uniformly distributed. We will show however, that the cell activation may follow an aggregation of a small fraction of the total amount of membrane receptors, with the total receptor amount unchanged. We will model the receptor aggregation by introducing the following modification to the spherically symmetric distribution of the receptors:
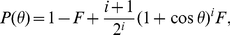
(13) where *F* describes the fraction of the aggregated receptors. For large *i* the aggregated receptors occupy approximately 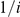 fraction of the total cell membrane. As shown in Figure 2B, for *F* = 0.01 and 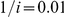, the trajectory of system (1)–(5) starting from the zero initial condition converges in time to the active steady state shown in Figure 2A. First, the cell activates itself in the vicinity of the receptor cluster; then the activation wave propagates around the whole cytoplasm. In this case the aggregation of 1% of the membrane receptors on the 1% fraction of the cell membrane causes that the local receptors density increases by a factor of 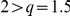. As a result, the system becomes locally monostable since, as discussed above, the *q*-fold increase of the receptor concentration leads to monostability. As in the rest of the membrane, the system remains bistable, the activated region may spread until the entire cytoplasm becomes active. In this scheme, there are two conditions necessary for cell activation:

- the global bistability, required for the wave front propagation
- the local monostability, required for the local activation and formation of the wave front.

Ad (I) The necessity of the global bistability for the full activation is demonstrated in Figure S3. The simulation is performed for the same parameters as chosen for Figure 2, but with the aggregated fraction of receptors *F* = 0.5. The system is locally active in the vicinity of the cluster, but since the large fraction of receptors is translocated from the rest of the membrane, the system is no longer globally bistable. Thus, the traveling wave cannot propagate. This shows that the local monostability of the system is not sufficient for the whole cell activation.

The global bistability does not imply that the wave front propagates in the “right” direction-i.e. that the active region grows. If the “energy” of the inactive state is lower than the “energy” of the active state, the active region will rather shrink than expand, see Figure S5. As a result, even if the system activates locally, the wave of kinase activity will not leave the region of the higher receptor concentration. The traveling wave propagates from inactive to active state when 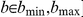 is close to 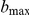. For 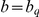 (defined in the previous section) the “energy” of the active state appears to be always smaller than the “energy” of inactive state and the wave propagates in the right direction.

In addition, the difference between the active and inactive state energies should be large enough to overcome the effect of the wave front curvature. Expansion of the curved wave front implies its elongation and growth of its energy-the effect is proportional to the value of the front curvature and thus it is important if the receptor cluster is small. In such a case the receptors may be activated locally, but the activity wave cannot propagate, see Text S1 for detailed analysis of the curvature effect. In Figure S4 we compare the conditions of straight and curved wave front propagation. In Figure S6 we show that aggregation of *F* = 0.1 fraction of receptors at 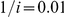 fraction of the cell surface leads to cell activation, but when the same fraction of receptors is clustered on 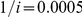 of cell surface, the cell activates only locally in the vicinity of the receptor cluster.

Ad (II) The system is locally monostable if the value of the local receptor concentration exceeds *q*, and the fraction of cell membrane occupied by the receptor cluster is large enough. For given α and *r_n_* there exists the minimal fraction of the cell surface 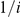 on which the receptor concentration must exceed *q* in order to achieve the local activation. In Figure 3, we show the critical fraction of the aggregated receptors 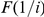 for α = 1, α = 3, α = 10 and for different values of nuclear radius *r_n_* = 0, 0.8, 0.9, 0.95 and 0.98. The critical fraction of the aggregated receptors decreases nearly linearly with the size of the cluster 1/*i* for sufficiently large 1/*i*. This is in accordance with the observation that for the sufficiently large cluster, cell activation is controlled by the local density of receptors in the cluster. This condition is well visible for small diffusion α = 10 and thin cytoplasm *r_n_* = 0.98, when the critical fraction of aggregated receptors *F* as a function of 1/*i* lies above but follows the line 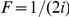. This implies that the local concentration of receptors must increase at least by *q* to trigger cell activation. However, when the size of receptor cluster 1/*i* becomes too small, this trend breaks. As shown in Figure S6, even if the cell activates locally, the front curvature is so large that its expansion is not possible. The further decrease of receptor cluster size causes that the cloud of the activated kinases is washed out by the diffusion and even the local activation is not possible. Thus, for any fixed *F* and 1/*i* sufficiently small the system does not activate even locally.

Summing up; for a given value of 1/*i* there exists the critical fraction of the aggregated receptors 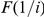 such that for 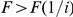 the cell activates. 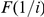 decreases with decreasing 1/*i* until 1/*i* achieves the limit value 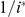, which corresponds to the (absolute) minimal fraction of receptors, 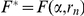, that must be aggregated in order to activate the cell. The smaller is the kinase diffusion (the larger value of α) the smaller is the wash-out effect and thus the fraction of aggregated receptors needed for activation is smaller, compare Figure 3 panels. Moreover, for fixed α, the fraction of aggregated receptors necessary for cell activation 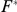 decreases monotonically with the thickness of cytoplasmic layer (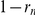), Figure 4. This is due to the fact that for thin cytoplasm the diffusion is rather 2D than 3D. As it is seen from Figure 4, for a given α such that 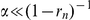, 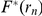 can be approximated by the formula 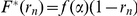. It is tempting to speculate that the thickness of cytoplasm provides a measure that distinguishes between activatory and nonactivatory clusters. For receptor clusters larger than the thickness of cytoplasm the diffusion of activated kinases is essentially 2D, which facilitates the wave front formation and propagation. As we will see in the next section the activatory cluster is much smaller under the assumption that kinases are confined to the cell membrane.

**Remark:** For a scalar reaction-diffusion equation
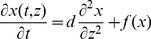
(14)the potential energy may be defined as a 
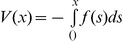
. In bistable scalar reaction-diffusion equation the direction of wave front velocity is such, that the region in which 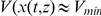, (where 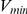 is the global potential minimum) expands, see e.g. [47]. As a result the total energy of the system decreases. For two-component system, the potential energy function 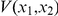 exists only when the source terms *f*_1_ and *f*_2_ satisfy, the consistency condition 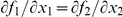. In this non-generic case, source terms can be expressed as potential derivatives i.e.
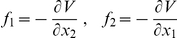



In our case the consistency condition is not satisfied, and thus our systems does not have a true potential. Therefore the term “energy” is used, only in the intuitive sense.

### Cell activation due to the displacement of the nucleus

Thinning of cytoplasm increases boundary to volume ratio and thus facilitates cell activation. As a result, the cell can be also activated by a displacement of the nucleus, which locally increases membrane to cytoplasm volume ratio. As shown in a related model by Meyers et al. [48], the increase of surface to volume ratio increases phosphorylation level. However, as opposed to Meyers et al. consideration, in our case the nucleus is not penetrated by the kinases. As a result only the cytoplasmic volume counts and thus the local cell activation may be induced without deformation of the plasma membrane. As shown in Figure 5, for *r_n_* = 0.9, α = 10, 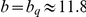 the cell activates in response to the displacement of the nucleus by 0.08, which causes that locally the thickness of the cytoplasm drops to 0.02. As a result, the cell locally activates, but interestingly, although the activity on the opposite pole is much lower, the activatory wave spreads also on the thicker parts of the cytoplasm. The displacement of the nucleus may accompany formation of the immunological synapse, when B cell scans the antigen presenting cell. It is well documented that in cytotoxic T lymphocytes, microtubule organizing center translocates towards target cell during synapse formation [43]. T cell polarization enables unidirectional killing. Although not reported, it is intuitive that the nuclear displacement takes place also in the early stage of B cell synapse formation when B cell spreads over antigen presenting cell, which requires cytoskeleton reorganization [11].

### Cell activation in the membrane model

In the membrane model the range of bistability in parameter *b*, [*b*_min_,*b*_max_], is the same as for the system in the infinite diffusion limit (10)–(11) and from the analytical examination of bistability of this system (see Figure S1), we have for *p* = 1, 
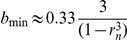
 and 
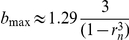
. The value of *b_q_*, as defined previously, is 
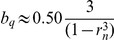
. In particular, we consider the membrane model corresponding to the cytosol model with 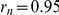, which gives the value of 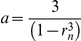. For this value of *a* we obtain 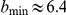, 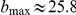, 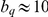.

The typical protein diffusion coefficients on membranes are about ten times smaller than in cytosol, accordingly the values of α expected in the membrane model are higher. For large α, as discussed in the Text S1 (see Figure S4), the effect of wave front curvature on wave propagation becomes negligible. In this limit, the front speed *c* can be approximated as 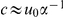, where *u*_0_ is the nondimensional coefficient characterizing the system (here 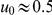). In dimensional units front speed is thus 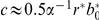, which gives the total cell activation time 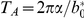, where, recall, we set 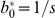. Wang et al. [41] found that mechanically induced wave of Src kinase activity propagates with velocity of 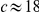
nm/s. Such propagation speed can be obtained in our model by taking α = 0.5×6 µm×(1/*s*)/(18 nm/s)≈167. For such α we obtained the cell activation time equal to *T_A_* = 1000 (see Figure 6). Obviously, the experiments of Wang et al. [41] performed on human umbilical vein endothelial cells may serve only as order of magnitude reference. The visual analysis of the data showed by Depoil et al. ([49], Figure 3) suggests that the wave of Syk kinase activity (which requires BCR activation) spreads outside the central cluster in time of order of 10 minutes. Collectively, these two experiments suggest that time scale of the activation process is of order of 10 minutes, which gives α within the range the experimental estimates, see Table 1.

In Figure 7 we compare 2D and 3D models for *r_n_* = 0.95 and α = 3 with respect to the dependence of the critical fraction of the aggregated receptors on the fraction 1/*i* of the cell surface occupied by the cluster. As in the 3D case, for any fixed *F* and 1/*i* sufficiently small the activation of the cell by the local receptor aggregation is not possible, even locally. As it is seen in Figure 7, the minimal fraction of the aggregated receptors 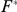 needed for activation in 2D case is much smaller than the minimal fraction of the aggregated receptors in the corresponding 3D case. For clusters occupying a large portion of cell surface, the critical fraction of aggregated receptors is almost the same for 3D and 2D models. When the diameter of the cluster becomes comparable with the thickness of the cytoplasmic layer, the activated kinase diffusion becomes 3D and the minimal clusters defined by the two models are different.

The minimum of activatory fraction 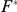 in 2D case decreases asymptotically as α^−2^ and for parameters 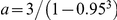, 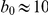 used for Figure 6, we numerically found that 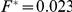 for α = 1; 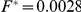 for α = 3; 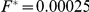 for α = 10. For 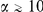 the minimal activatory fractions of receptors predicted by the membrane model is less than 10^−3^. Since the estimates based on dephosphorylation and diffusion constants suggest that α>10, one can expect that the minimal size of the activatory aggregate is determined by the magnitude of the stochastic fluctuations. As found by Dintzis and Vogelstein, BCRs aggregates of ten or more receptors are signaling competent [18], [19]. Aggregates smaller than ten BCRs possibly switch *ON* and *OFF* too fast to trigger traveling waves. However, when *OFF* rate is small as in the example of Ras activation considered in [50] even the local stochastic fluctuation of kinase activity can initiate travelling wave propagation.

## Discussion

The spatiotemporal kinetics of proteins and other substrates regulate cell fate and signaling [51]–[52]. The temporal dynamics is coupled with spatial gradients of concentrations or activity. The gradients of kinase activity come about when phosphorylation and dephosphorylation proceed at different cellular locations. Here, we proposed a spatially-extended B cell activation model exploiting the intrinsic chemical and geometrical properties of the system:

1. positive feedback coupling activation between B cell receptors and Src family kinases which, together with the nonlinearities due to the double receptor phosphorylation and the Michaelis-Menten dephosphorylation kinetics, leads the system to bistability. The studies of Fuss et al. [38], [39] and Kaimachnikov and Kholodenko [40] showed that SFK can display bistability. Bistability in the SFK-BCR system can be expected since B cell activation dramatically changes its function initiating B cell transformation into an antibody secreting cell or a memory cell. The cell fate decisions are thought to be controlled by bi- or multistable regulatory systems.
2. B cell has a very thin cytoplasm which increases the concentration of kinase molecules and limits their diffusion. Moreover, most of Src family kinases are tethered to the cellular membrane which further restricts their motility. The cross-linking of receptors due to their binding to polyvalent ligands practically immobilizes them, which allows us to assume that in short time scale the concentration of receptors 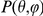 is constant.

B cells can be activated by the formation of a relatively small receptor cluster that consists of a small fraction of the total number of receptors. This suggests that such a cluster, or clusters, can serve as a switch triggering the response which is not proportional to the signal. These features of B cell activation bear some similarities to the process of calcium wave initiation and propagation. The traveling wave of free calcium concentration in the cytosol can be induced by a sufficient number of strongly localized Ca^2+^ ions released from the internal stores (endo- or sarcoplasmic reticulum) to the cytosol [53]. The released calcium interacts with the ryanodine receptors located on the surface of the internal stores leading to the further calcium release, which provides positive feedback to the process.

As found by Dintzis and Vogelstein, the interconnection of ten or more BCRs in one cluster (immunon) due to binding of highly polyvalent antigens is sufficient for emitting an immunogenic signal [18], [19]. This characterizes the ‘immunon’ as a quantum of aggregated receptors necessary for delivering an immunogenic signal. The immunon hypothesis was then developed into a mathematical theory by Perelson and Sulzer [21]. Though this theory takes into account only some aspects of very complicated phenomena [54], it proved to be successful in explaining the dose-response curves obtained by Dintzis et al. [55]. The alternative way of formation of BCR clusters is based on spreading and contraction of B cell on antigen presenting cells, which selectively sequesters high affinity antigens to a small fraction of B cell membrane (reviewed in [1]). In this mode of activation the receptor aggregate can be formed even if the antigens are monovalent. This finding has demonstrated that although local receptor aggregation is necessary for triggering B cell activation, the physical cross-linking of receptors by antigens is dispensable.

In our study we focus on the spatial aspects of B cell receptor signaling, keeping the chemical reaction part simplified. We expect, however, that inclusion of the omitted details of the chemical interactions would not qualitatively influence the overall dynamics, provided that the full system retains bistability.

Within the proposed model we demonstrated that displacement of the nucleus, and the resulting local thinning of the cytoplasmic layer can trigger local BCR and SFK activation. The activity wave can then propagate throughout the rest of membrane and cytoplasmic layer. The activation is induced by the locally increased ratio of the membrane surface to the cytoplasm volume, and the fact that nucleus is not penetrated by the Src kinases. The nuclear movements during synapse formation are well documented experimentally in T lymphocytes [43]. Due to the fact that the B c,ell cytoplasm layer is uniformly thin, this effect was, to the authors' knowledge, not reported for B lymphocytes. However, it is intuitive that the local narrowing of the cytoplasmic layer does take place in the early stage of immunological synapse formation when B cell spreads over APC, possibly by means of cytoskeleton reorganization as reported by [11]. Such local narrowing of the cytoplasmic layer would facilitate B cell activation.

A similar activation effect due to the change of geometry was discussed by Meyers and colleagues [48], who showed that cell flattening at constant volume increases global phosphorylation levels [48]. However, since Meyers et al. assumed that the nucleus can be penetrated by Cdc42 protein, they found that Cdc42 activation should be greatest where the cell is thinnest, which is generally the case near the edges of the cell, and lowest where the cell is thickest, which is generally near the nucleus. The local increase of the membrane surface to cell volume ratio can be also due to membrane ruffling; Onsum et al. showed that asymmetry in membrane distribution due to ruffling leads to the amplified internal gradient of phosphatidylinositol-3,4,5-triphosphate [56]. Interestingly, cell signaling can be also induced by deformation of extracellular matrix [57], [58]. In particular, Maly et al. [57] showed that autocrine EGFR signalling can be induced directly by the mechanical deformation of the tissue leading to several fold increase of the ligand concentration.

Our main result is the demonstration that the local receptor activation due to the formation of a small receptor cluster is sufficient for triggering activation of the remaining receptors. We showed that the critical amount of the aggregated receptors required for cell activation decreases with the thickness of the cytoplasm. This effect is different than the effect discussed above (and analyzed by Meyers et al. [48]) where the kinase activity grows with decreasing thickness of the cytoplasmic layer. In accordance with this finding, in our model the bistability range in dephosphorylation coefficient *b*, shifts to the larger values as the thickness of the cytoplasm decreases. To adjust to this effect we assume that the system (for each value of *r_n_*) is in the bistability range, and that the actual value of *b* (which in real cells is governed by the amount of phosphatase) is such that the receptor density must grow by 50% in order to move the system to the active monostable regime. Then, under this assumption we have examined the dependence of the minimal activatory cluster on the thickness of the cytoplasm. As it is clear from Figures 3 and 4, the size of the minimal cluster is decreasing with the decreasing thickness of the cytoplasm. This can be explained as follows: the receptor density within the cluster must be sufficiently large to induce the local activation. When, in addition, the diameter of the cluster is larger than the thickness of the cytoplasm, the diffusion of activated kinases is essentially two, rather than three, dimensional, thus facilitating wave propagation. Heuristically, we can say that the thickness of the cytoplasm (which for B cells is about 0.4 µm) is a geometric measure used by the cell to decide whether the cluster of receptors is sufficiently large.

As already stated, most of SFK, in contrast to cytosolic Syk kinase, is predominantly tethered to the plasma membrane. Since SFK and Syk mediate positive feedback, we considered also the alternative model in which we assumed that kinase diffusion is restricted to the membrane. In fact, we would expect the membrane model to be more biologically justified because SFK-BCR system alone exhibits bistability necessary for wave propagation. Comparing the membrane and cytoplasmic models (see Figure 7), we found that the minimal activatory cluster is even smaller under the assumption that the kinases are confined to the cell membrane. Relevant to the membrane model, Wang, et al. [41] found that mechanically induced wave of Src kinase activity propagates on the membrane of vein endothelial cells with the speed of *c* = 18 nm/s, which corresponds to the cell activation time of 1000 s (assuming cell radius of 6 μm). To reproduce this propagation speed we set α = 167, which falls within the experimentally established range (see Table 1). Also the measurements of the kinase activity induced by formation of BCR-antigen microclusters indicated that within 10 min after receptor aggregation, Syk activity spreads outside the central supramolecular activation cluster (cSMAC) [49]. It would be interesting to verify experimentally, whether the wave of Syk and SFK kinases activity manages to propagate to the opposite (to cSMAC) pole of the cell.

## Supporting Information

Figure S1Bistability range in (*B*,*H*) plane for the spatially uniform model. The points situated between the hyperbola-like curves of the same color correspond to the bistable regime for the given *c*_0_. For *c*_0_ = 0 the bistability region extends from the *H*-axis and *B*-axis to the blue hyperbola-like curve. For *c*_0_ = 0.01 and *H* = 0.1 assumed for this study bistability range (for the spatially uniform model) in *B* parameter is 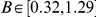.(TIF)

Figure S2Bistability and monostability regions in (*b*,*p*) plane for 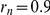, *c*_0_ = 0.01, 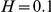, and three values of α; α = 0, α = 3, α = 10.(TIF)

Figure S3Global bistability is required for the activatory wave propagation. The steady state solution for α = 10, 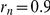, 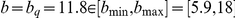 with the aggregated fraction is 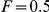 (50% receptors aggregated close to the pole) and cluster size 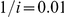. Due to the large fraction of the aggregated receptors the system is not globally bistable and the traveling wave cannot propagate. The traveling wave propagates in the simulation performed with same parameters but with the smaller fraction of the aggregated receptors equal *F* = 0.01, as shown in Figure 2 (in the main document).(TIF)

Figure S4Analysis of the wave front curvature effect. Left panel: standing wave solutions 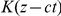 to system (2)-(3) for α = 1, α = 3, α = 10 and 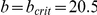. Middle panel: stationary solution for *i* = 10000, *F* = 0.1 and α = 1, α = 3, α = 10 and corresponding critical values of *b* (above which the wave cannot propagate), *b_crit_* = 16.3, 17.6 and 19, respectively. Right panel: stationary solution for *i* = 10000, *F* = 0.1 and α = 1, α = 3, α = 10 and corresponding critical values of *b*, *b_crit_* = 14.4, 15.8 and 17.8, respectively.(TIF)

Figure S5Travelling wave propagates from the inactive to the active state. For α = 10, 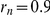, 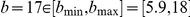 (i.e. close to *b*_max_), the “energy” of the inactive state is lower than the energy of the active and the activity wave propagates backward, i.e. the inactive region grows until entire cell becomes inactive.(TIF)

Figure S6The effect of the front curvature analyzed for α = 10, 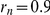, 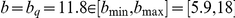. Panel A: for the aggregated fraction 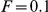 and cluster size 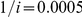 the system activates locally, but the wave cannot propagate because the curvature of the wave front is too large; Panel B: wave front propagates for a larger receptor cluster 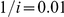 with the same aggregated fraction 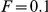.(TIF)

Text S1 This supplementary text includes six figures and is divided into two sections: *System bistability analysis* and *Conditions for the activatory traveling wave propagation.* In the first section, we analyze the conditions guaranteeing the bistability of the system both for spatially homogeneous system and the system with finite diffusion. In the second section, we analyze the influence of the front curvature on propagation of activatory traveling waves.
(PDF)
