# Supplementary figures and images for: B Cell Activation Triggered by the Formation of the Small Receptor Cluster: A Computational Study
Source: PLoS Comput Biol. 2011 Oct 6;7(10):e1002197. doi: 10.1371/journal.pcbi.1002197 (PMC3188507; doi:10.1371/journal.pcbi.1002197)

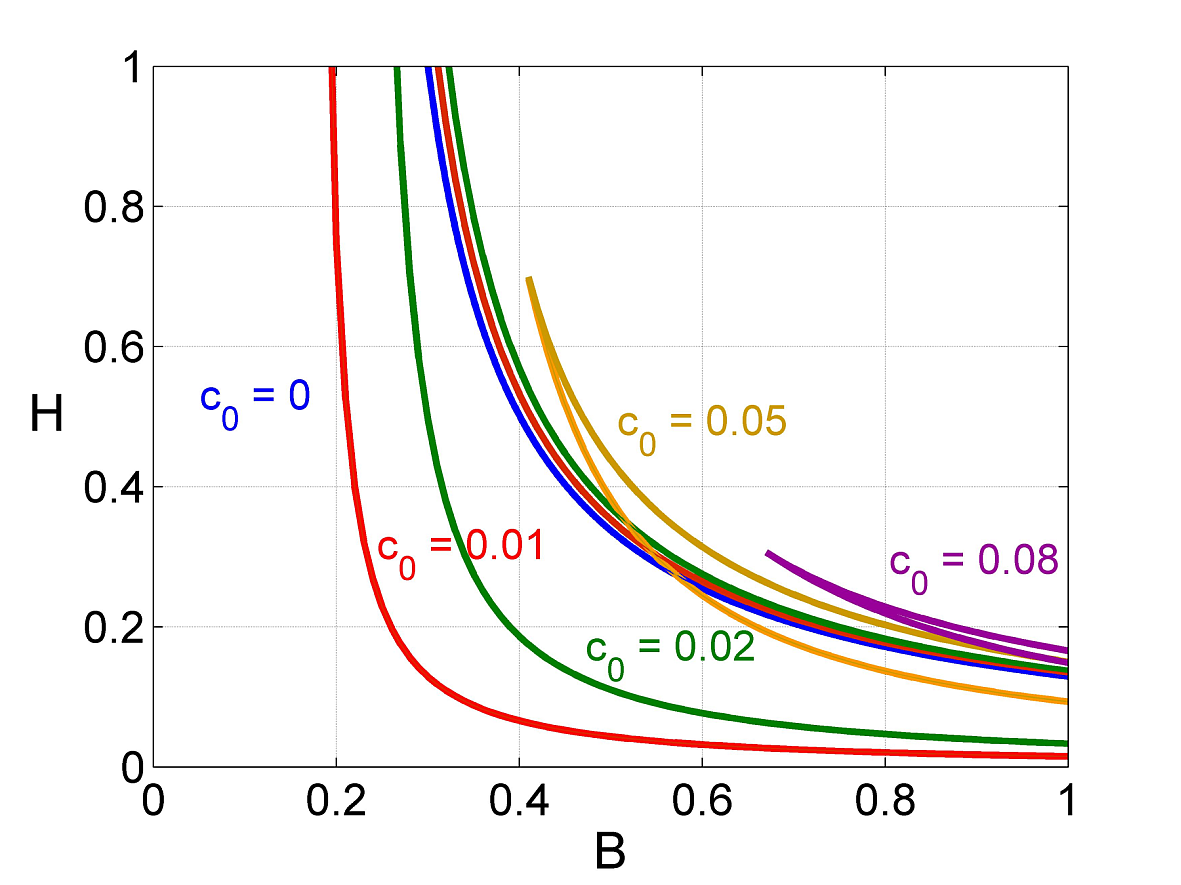

Supplement: Figure S1 — Bistability range in (B,H) plane for the spatially uniform model. The points situated between the hyperbola-like curves of the same color correspond to the bistable regime for the given c 0. For c 0 = 0 the bistability region extends from the H-axis and B-axis to the blue hyperbola-like curve. For c 0 = 0.01 and H = 0.1 assumed for this study bistability range (for the spatially uniform model) in B parameter is . (TIF) [file pcbi.1002197.s001.tif]

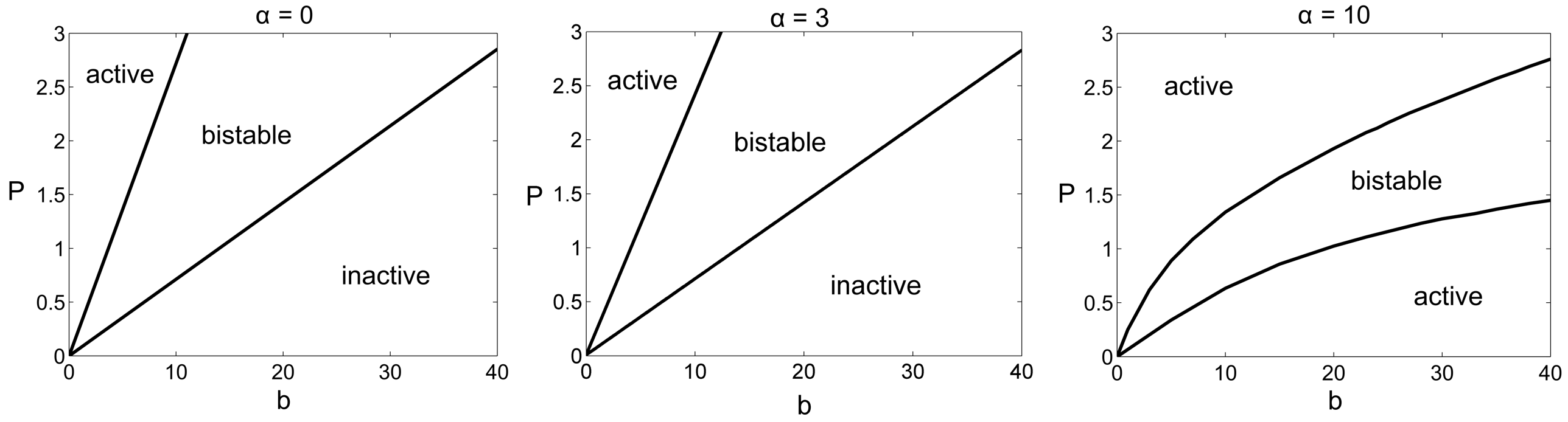

Supplement: Figure S2 — Bistability and monostability regions in (b,p) plane for , c 0 = 0.01, , and three values of α; α = 0, α = 3, α = 10. (TIF) [file pcbi.1002197.s002.tif]

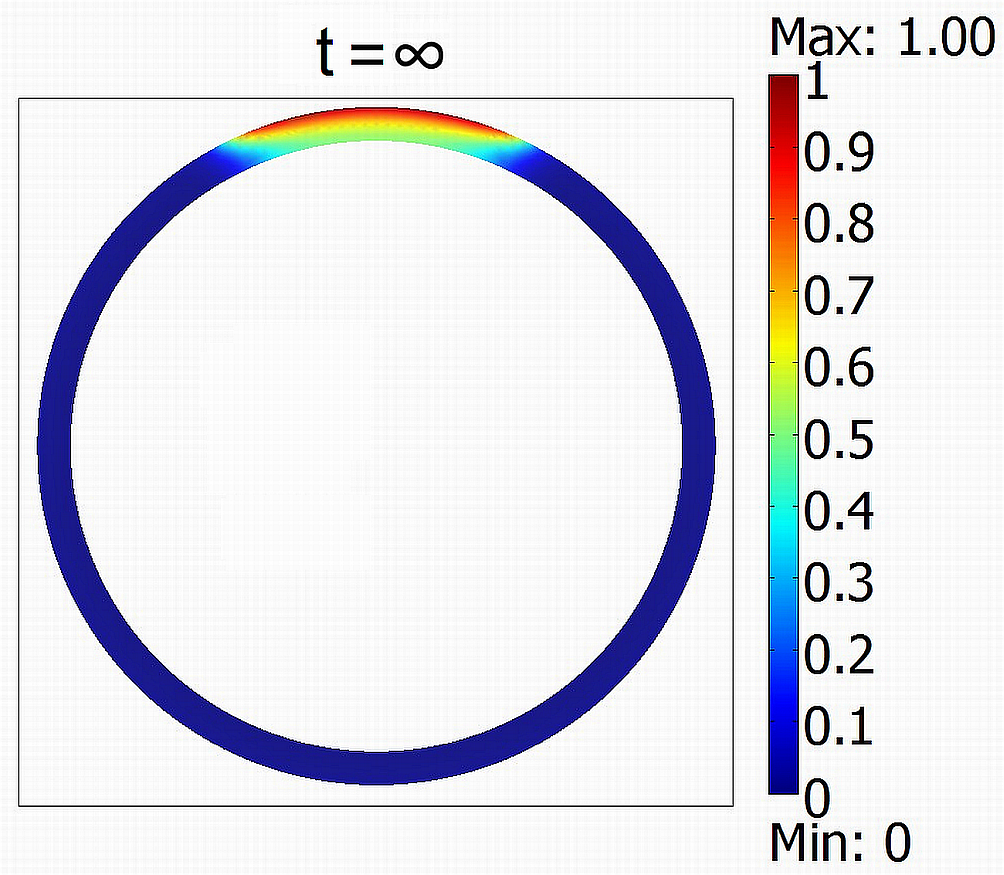

Supplement: Figure S3 — Global bistability is required for the activatory wave propagation. The steady state solution for α = 10, , with the aggregated fraction is (50% receptors aggregated close to the pole) and cluster size . Due to the large fraction of the aggregated receptors the system is not globally bistable and the traveling wave cannot propagate. The traveling wave propagates in the simulation performed with same parameters but with the smaller fraction of the aggregated receptors equal F = 0.01, as shown in Figure 2 (in the main document). (TIF) [file pcbi.1002197.s003.tif]

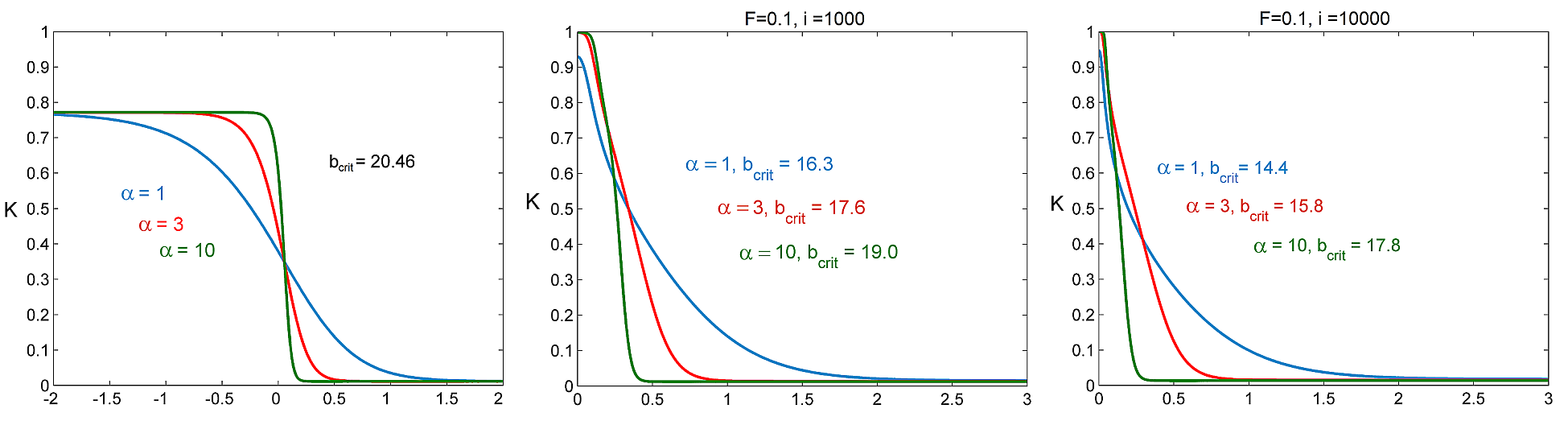

Supplement: Figure S4 — Analysis of the wave front curvature effect. Left panel: standing wave solutions to system (2)-(3) for α = 1, α = 3, α = 10 and . Middle panel: stationary solution for i = 10000, F = 0.1 and α = 1, α = 3, α = 10 and corresponding critical values of b (above which the wave cannot propagate), bcrit = 16.3, 17.6 and 19, respectively. Right panel: stationary solution for i = 10000, F = 0.1 and α = 1, α = 3, α = 10 and corresponding critical values of b, bcrit = 14.4, 15.8 and 17.8, respectively. (TIF) [file pcbi.1002197.s004.tif]

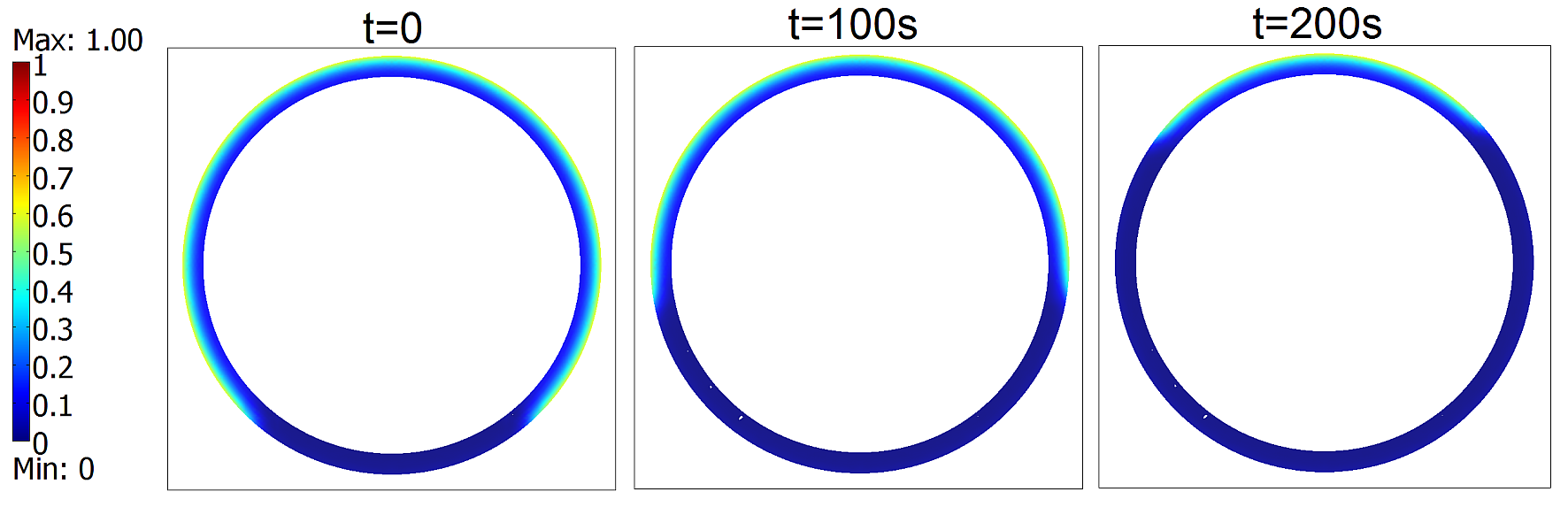

Supplement: Figure S5 — Travelling wave propagates from the inactive to the active state. For α = 10, , (i.e. close to b max), the “energy” of the inactive state is lower than the energy of the active and the activity wave propagates backward, i.e. the inactive region grows until entire cell becomes inactive. (TIF) [file pcbi.1002197.s005.tif]

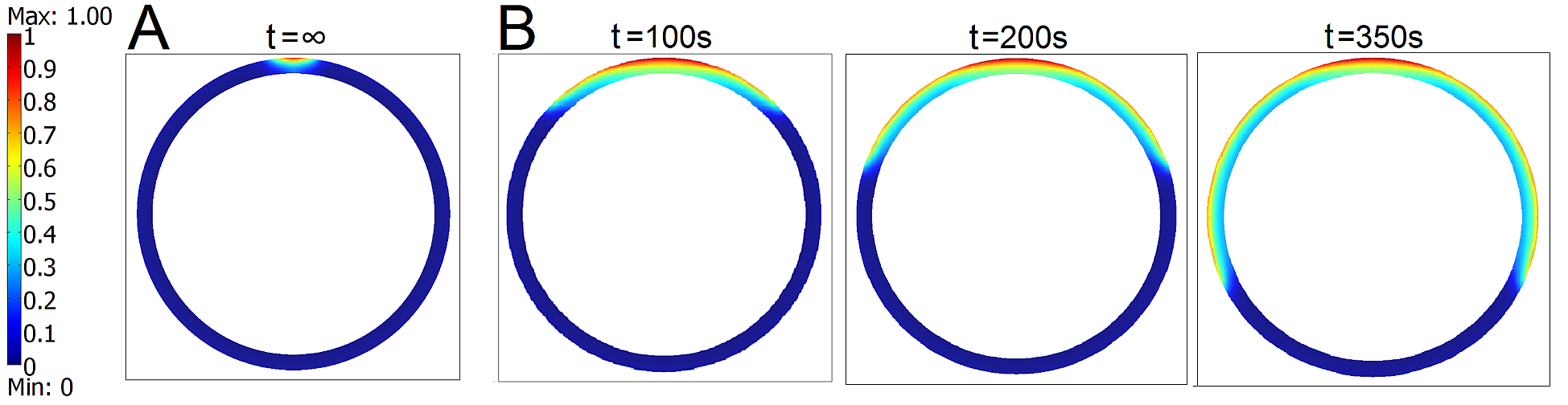

Supplement: Figure S6 — The effect of the front curvature analyzed for α = 10, , . Panel A: for the aggregated fraction and cluster size the system activates locally, but the wave cannot propagate because the curvature of the wave front is too large; Panel B: wave front propagates for a larger receptor cluster with the same aggregated fraction . (TIF) [file pcbi.1002197.s006.tif]
