# Supplementary material for: B Cell Activation Triggered by the Formation of the Small Receptor Cluster: A Computational Study
Source: PLoS Comput Biol. 2011 Oct 6;7(10):e1002197. doi: 10.1371/journal.pcbi.1002197 (PMC3188507; doi:10.1371/journal.pcbi.1002197)
Supplement: Text S1 — This supplementary text includes six figures and is divided into two sections: System bistability analysis and Conditions for the activatory traveling wave propagation. In the first section, we analyze the conditions guaranteeing the bistability of the system both for spatially homogeneous system and the system with finite diffusion. In the second section, we analyze the influence of the front curvature on propagation of activatory traveling waves. (PDF) [file pcbi.1002197.s007.pdf]

Supplementary Material for "B Cell Activation Triggered by the Formation of the Small Receptor Cluster: a Computational Study"

Beata Hat, Bogdan Kazmierczak, Tomasz Lipniacki

I. System bistability analysis

The bistability of the model arises due to the nonlinearities in kinases dephosphorylation (Michaelis-Menten dephosphorylation kinetics) and second order nonlinearity in receptor activation due to double phosphorylation. Each of these two nonlinearities alone, suffices for the bistability. The contribution of these two nonlinearities is controlled by Michaelis constant H (the larger is the value of H the more linear is dephosphorylation kinetics) and the value of c_0 (the larger is c_0 the more linear is the receptor activation function $c_0 + K^2$ since always $K < 1$). In fact, there are additional nonlinearities in the system, that is, nonlinearity in receptor dephosphorylation, and kinase transphosphorylation. These nonlinearities are omitted for the sake of simplicity. It is natural to expect that inclusion of these nonlinearities would make the bistability region broader.

First, we confine ourselves to spatially uniform solutions ($\alpha = 0$) and analyze the dependence of the bistability regions in the (B, H) plane on the coefficient c_0. Using Eqs. (10),(11) (from the main text) we infer that the constant steady state solutions are given by

the solutions of the system:

$$R(1-K) - \frac{BHK}{H+K} = 0 \tag{1}$$

$$(c_0 + K^2)(P-R) - R = 0. \tag{2}$$

which for $c_0 = 0$ simplifies to

$$R(1-K) - \frac{BHK}{H+K} = 0, \quad K^2(P-R) - R = 0. \tag{3}$$

It follows that for $c_0 = 0$ and for any $B > 0$ and $H > 0$ system (3) has a zero solution $K = 0$, $R = 0$. This solution is stable, because the Jacobian matrix J_0 of the vector function $(R(1-K) - \frac{BHK}{H+K}, K^2(P-R) - R)$ calculated for $K = 0$, $R = 0$ has two negative eigenvalues equal to -1 and $-B$. In fact, J_0 is the linearization matrix of system (10)-(11) (main document) around the zero solution $(K, R) = (0, 0)$.

The stable positive solution arises when the value of B is sufficiently small. As a result for $c_0 = 0$, the bistability region (Figure S1) extends from the lines $B = 0$ and $H = 0$ to the line $B_R(H)$ at which the (stable) positive solution vanishes. For $c_0 > 0$, there is no zero solution and the bistability region becomes separated from $B = 0$ and $H = 0$ lines. For $c_0 > 0$ the bistability region is bounded from the left by line $B_L(H)$ at which the "inactive solution" arises and from the right by line $B_R(H)$ at which the "active" solution vanishes. Since the system behavior for $c_0 = 0$ is not representative, we choose $c_0 = 0.01$, which is approximately equivalent to the assumption that the activity of the unphosphorylated kinase is 100 times smaller than the activity of phosphorylated kinase.

Next, we set $r_n = 0.9$, $c_0 = 0.01$, $H = 0.1$ and analyze the bistablity regions in (b, P) plane for three values of α. As shown in Figure S2 the bistability range in (b, P) plane for $\alpha = 3$ is almost identical to the bistability range for the infinite diffusion, $\alpha = 0$. This shows that the analysis presented in Figure S1 can serve as a rough reference also for the finite but large diffusion α^{-2}.

II. Conditions for activatory traveling wave propagation

The traveling waves observed numerically both in the cytosolic and the membrane models are not the usual plane traveling waves. In the cytosolic model, at a given moment of time, the values of K and R are not uniform along the radius direction. In both models the curvature of the wave front is very important - especially when the activation starts from receptors cluster occupying a small fraction of the cell membrane. Here, basing on the membrane model, we will discuss the necessary conditions for activatory traveling wave propagation, then we will provide some numerical examples (Figures S5 and S6) for the cytoplasmic model for which the analysis is more complicated.

In the membrane model the equation for the active kinase concentration K may be written as

$$\frac{\partial K\,(t,\theta)}{\partial t} = \alpha^{-2}\ \cot\theta\ \frac{\partial K}{\partial \theta}\ + \alpha^{-2}\ \frac{\partial^2 K}{\partial \theta^2}\ + aR(1-K) - \frac{bHK}{H+K}, \tag{4}$$

The first term of the right-hand-side of Eq. (4) is associated with the wave front curvature. Presence of the curvature term causes that the wave front propagation velocity depends on θ. As shown in Figure 7 (in the main document) in the main document, the velocity of activatory front increases with θ. Without the curvature term the "membrane model" (for $a = 1, P = 1$) reads

$$\frac{\partial K\,(t,z)}{\partial t} = \alpha^{-2}\ \frac{\partial^2 K}{\partial z^2}\ + R(1-K) - \frac{bHK}{H+K}, \tag{5}$$

$$\frac{dR\,(t,z)}{dt} = (c_0 + K^2)(1-R) - R, \tag{6}$$

and may be considered on the infinite domain $z \in (-\infty, \infty)$. It has thus traveling wave solutions

$$K\,(t,z) = K(z-ct), \qquad R\,(t,z) = R(z-ct). \tag{7}$$

In this case, the propagation velocity c is $c = \alpha^{-1}\ f(b, H, c_0)$. For fixed $H = 0.1, c_0 = 0.01$, the system is bistable for $b \in [6.73, 27.1]$ and the bistability range is independent of α. The

critical value of b (b_{crit}) corresponds to standing wave solutions with $c = 0$. For $b < b_{crit}$ the activatory traveling waves may propagate, while for $b > b_{crit}$, traveling waves propagate in the opposite direction - thus they can be considered de-activatory. For the reduced system (5)-(6) $b_{crit} = 20.5$.

The sign of the curvature term $\alpha^{-2}\cot(\theta)\partial K/\partial\theta$, for monotone traveling wave profile ($\partial K/\partial\theta < 0$, see Figure 7 in the main document), is determined by the sign of the function $\cot(\theta)$. For $\theta < \pi/2$ presence of the curvature term decreases $\partial K/\partial t$ and thus traveling wave velocity (assuming that the wave propagates from $\theta = 0$ to $\theta = \pi$), while for $\theta > \pi/2$ the curvature term increases the propagation velocity. For small values of θ, the contribution of the curvature term may dominate the right-hand-side of Eq. (4) and change the sign of the wave front propagation, or prevents the front from leaving the receptors cluster area in which R is large. One may thus expect that the critical value of b, below which the activatory wave will leave the receptor cluster will be smaller than the critical value of b found for plane front, $b_{crit} = 20.5$. In order to demonstrate the curvature effect we consider the membrane model with the following receptor distribution

$$P(\theta) = 1 + \frac{i+1}{2^i}(1+\cos\theta)^i F$$

and calculate b_{crit} for two values of i, $i = 1000$ and $i = 10000$ and three values of α. The large value of $F = 0.1$ implies the local system activation (see Figure S4) but does not imply that activatory wave front will propagate over the area where the receptors density is close to 1. For $i = 1000$ we obtained the critical values of the parameter b, as 16.3, 17.6 and 19 for $\alpha = 1$, $\alpha = 3$ and $\alpha = 10$ respectively. For $i = 10000$, b_{crit} is equal 14.4, 15.8 and 17.8 for $\alpha = 1$, $\alpha = 3$ and $\alpha = 10$ respectively. The curvature effect, i.e. the difference between the critical value of b, $b_{crit} = 20.5$, obtained for plane traveling waves and b_{crit} for the membrane model (5)-(6), increases with increasing i (i.e. initial front curvature) and with decreasing α. The curvature effect causes that the clusters of very small area are not activatory.
